# Predictors of Successful Yttrium-90 Radioembolization Bridging or Downstaging in Patients with Hepatocellular Carcinoma

**DOI:** 10.1155/2021/9926704

**Published:** 2021-07-23

**Authors:** Alexander Villalobos, William Wagstaff, Mian Guo, James Zhang, Zachary Bercu, Morgan J. Whitmore, Mircea M Cristescu, Bill S. Majdalany, Joel Wedd, Mehmet Akce, Joseph Magliocca, Marty T. Sellers, Nima Kokabi

**Affiliations:** ^1^Department of Radiology and Imaging Sciences, Emory University School of Medicine, Atlanta, GA, USA; ^2^Department of Biostatistics and Bioinformatics, Rollins School of Public Health, Emory University, Atlanta, GA, USA; ^3^Division of Digestive Diseases, Department of Medicine, Emory University School of Medicine, Atlanta, GA, USA; ^4^Department of Hematology and Medical Oncology, Emory University School of Medicine, Atlanta, GA, USA; ^5^Division of Transplantation, Department of Surgery, Emory University School of Medicine, Atlanta, GA, USA

## Abstract

**Purpose:**

This study aims to identify clinical and imaging prognosticators associated with the successful bridging or downstaging to liver transplantation (LT) in patients undergoing Yttrium-90 radioembolization (Y90-RE) for hepatocellular carcinoma (HCC).

**Methods:**

Retrospectively, patients with Y90-RE naïve HCC who were candidates or potential candidates for LT and underwent Y90-RE were included. Patients were then divided into favorable (maintained or achieved Milan criteria (MC) eligibility) or unfavorable (lost eligibility or unchanged MC ineligibility) cohorts based on changes to their MC eligibility after Y90-RE. Penalized logistic regression analysis was performed to identify the significant baseline prognosticators.

**Results:**

Between 2013 and 2018, 135 patients underwent Y90-RE treatment. Among the 59 (42%) patients within MC, LT eligibility was maintained in 49 (83%) and lost in 10 (17%) patients. Within the 76 (56%) patients outside MC, eligibility was achieved in 32 (42%) and unchanged in 44 (58%). Among the 81 (60%) patients with a favorable response, 16 (20%) went on to receive LT. Analysis of the baseline characteristics revealed that lower Albumin-Bilirubin score, lower Child–Pugh class, lower Barcelona Clinic Liver Cancer stage, HCC diagnosis using dynamic contrast-enhanced imaging on CT or MRI, normal/higher albumin levels, decreased severity of tumor burden, left lobe HCC disease, and absence of HBV-associated cirrhosis, baseline abdominal pain, or fatigue were all associated with a higher likelihood of bridging or downstaging to LT eligibility (*p*'s < 0.05).

**Conclusion:**

Certain baseline clinical and tumor characteristics are associated with the successful bridging or downstaging of potential LT candidates with HCC undergoing Y90-RE.

## 1. Introduction

Liver cancer remains the fourth leading cause of malignancy-related mortality worldwide [1], with a 5-year survival of 15% and a lethality profile second only to pancreatic cancer [2]. Globally, the complexity of the chronic liver disease patient and the heterogeneity of hepatocellular carcinoma (HCC) continue to challenge the development of effective treatments. Nevertheless, many therapies exist, with liver transplantation (LT) remaining as the gold standard for its ability to treat the malignancy and the often present underlying cirrhosis simultaneously.

The opportunity for a patient to attain LT is limited, especially for those residing in geographical regions where donor organ shortages exist or for those with tumors outside accepted transplant candidacy criteria [3]. To address this challenge, many transplant centers have embraced bridging or downstaging therapies, which are, respectively, aimed at ameliorating tumor progression or reducing tumor burden [4].

Current National Comprehensive Cancer Network and American Association for the Study of Liver Diseases (AASLD) guidelines state that patients deemed candidates or potential candidates for LT, as per multidisciplinary evaluation, should be evaluated for bridging or downstaging therapy at select institutions [5, 6]. Only recently has the use of Yttrium-90 radioembolization (Y90-RE) been evaluated for the purposes of providing bridging or downstaging therapy [7–9]. In light of these contemporary changes, the aim of this study was to identify clinical and imaging prognosticators associated with the successful bridging or downstaging to LT candidacy, as defined by Milan criteria (MC), in patients with HCC undergoing Y90-RE therapy.

## 2. Materials and Methods

### 2.1. Study Design

With institutional review board approval and HIPAA compliance, a single tertiary center retrospective study was performed at a high-volume academic transplant institution. An initial cohort of patients was identified who had successfully undergone Y90-RE for the treatment of HCC. To be included in the study, patients had to be at least 18 years of age, have complete pre- and post-Y90-RE clinical and imaging records, and be found to be a potential LT candidate by the institution's multidisciplinary tumor board (MTB). Because of the increasing evidence of Y90-RE's ability to outperform transarterial chemoembolization (TACE) and ablation [10–12], the study's institution considers Y90-RE as first-line generally whenever a LT eligible or potentially eligible patient has total bilirubin of <2 mg/dL and albumin of >3 g/dL. While it has been previously reported that worsening hepatic function can occur after Y90-RE irrespective of the BCLC stage [13,14], recent literature has found that most incidences of post-Y90-RE hepatic dysfunction are self-limited and of minimal to no clinical significance [15]. Nevertheless, many institutions, including the one for this study, often select patients with borderline hepatic function to undergo Y90-RE based on widely accepted, relatively conservative criteria that aim to balance the estimated risk of post-Y90-RE hepatic dysfunction with the expected potential survival benefit [16]. Accordingly, patients with Child–Pugh (CP) class B8 or greater were considered as being beyond criteria to receive any Y90-RE. Diagnosis of HCC was determined by either tissue sampling or characteristic imaging findings on dynamic contras-enhanced (DCE) CT or MRI. Imaging tumor response to Y90-RE therapy was retrospectively evaluated by an abdominal fellowship-trained radiologist with at least 5 years of experience using the modified Response Evaluation Criteria in Solid Tumors (mRECIST) criteria for HCC treatment evaluation in accordance with AASLD guidelines [17]. Because of picture archiving and communication system searching capability limitations, only patients who received Y90-RE therapy after 2013 were able to have their complete imaging data captured and reviewed.

**Figure 1:**
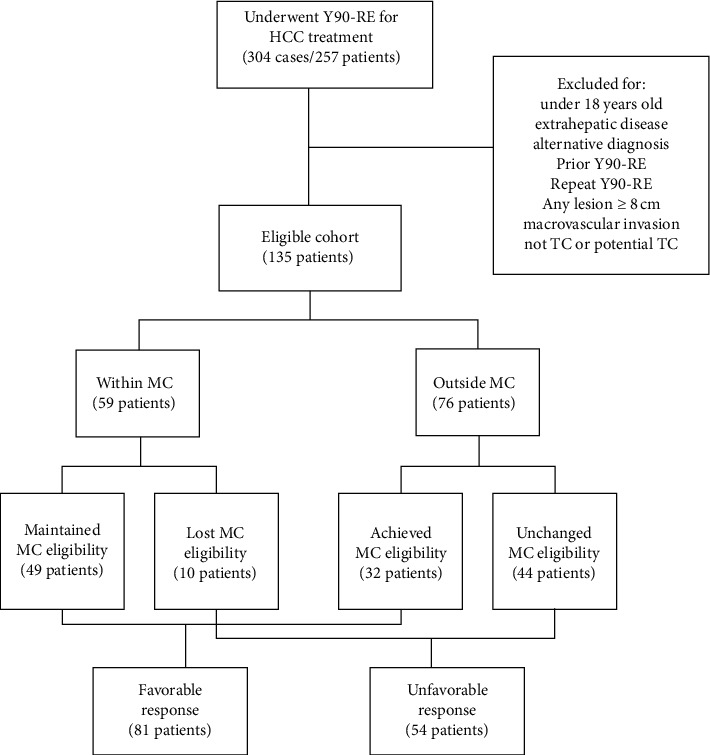
Cohort flowchart. MC = Milan criteria; HCC = hepatocellular carcinoma; TC = transplant candidate; Y90-RE = Yttrium-90 radioembolization.

Patients were excluded from analysis if they were not deemed a transplant candidate or potential transplant candidate by MC (single tumor ≤ 5 cm, or up to three ≤ 3 cm tumors) at the time of MTB evaluation. Patients were also excluded if they had macrovascular invasion, extrahepatic disease, alternative diagnoses discovered on liver tissue sampling (e.g., cholangiocarcinoma or mixed HCC-cholangiocarcinoma), or any Y90-RE therapies prior to evaluation and referral from the MTB. Considering literature suggesting worse outcomes for patients with single HCC tumors measuring greater than 8 cm in diameter [18], patients with any tumor diameter greater than 8 cm were also excluded (Figure 1).

Patients who underwent Y90-RE as bridging or downstaging therapy were divided into favorable or unfavorable Y90-RE response groups based on changes to their MC eligibility. A favorable response to Y90-RE was defined as maintaining or achieving MC eligibility, while an unfavorable response was defined as losing MC eligibility or remaining MC ineligible.

Clinical data were obtained using the baseline pre-Y90-RE evaluation and 30 days post-Y90-RE therapy. Collected clinical data included patient demographics, baseline laboratory and clinical history, and pre- and posttreatment symptomatology. Captured cancer-related characteristics included liver disease etiology, prior HCC treatments, method of HCC diagnosis, and effect on MC eligibility. HCC imaging characteristics were obtained from pretreatment (30 days prior to treatment) and posttreatment (up to 90 days) DCE liver protocol MRI or CT imaging. Imaging findings included tumor lobe distribution, portal vein invasion, portal vein thrombosis, extrahepatic involvement, and tumor sizes for the three largest tumors.

Other clinical variables such as the Model for End-Stage Liver Disease with Sodium (MELD) score, Albumin-Bilirubin (ALBI) grade [19], Albumin-to-Alkaline Phosphatase Ratio (AAPR) [20], CP class, Barcelona Clinic Liver Cancer (BCLC) stage, and Eastern Cooperative Oncology Group (ECOG) performance scores were included from the routine pre- and posttherapy evaluation. Where appropriate, clinical and laboratory adverse events (AE) were graded in accordance with the Common Terminology Criteria for Adverse Events v5.0 [21]. Decisions regarding the Y90-RE treatment strategies, such as dose amount and targeting technique (e.g., segmentectomy vs. lobectomy), were made at the discretion of the interventional radiologists and the planning nuclear medicine physician at the time of consultation and treatment planning. Because of the potential for LT and the study institution's recognition of the tumor dose-response relationship [22], all patients in this study underwent planning to receive the highest tolerable tumor dose and were treated with aggressive curative intent (not with palliative intent).

### 2.2. Radioembolization Procedure

Y90-RE was performed in accordance with previously described methods [23, 24]. Briefly, patients first underwent a shunt study to evaluate the mesenteric, extrahepatic, and intrahepatic vasculature prior to Y90 administration. During this study, Technetium-99m macroaggregated albumin was injected into the lobar or segmental hepatic artery supplying the targeted tumors to confirm complete coverage of the tumor. Patients then underwent a planar and single-photon emission computed tomography (CT) shunt study to determine the lung shunt fraction and any potential extrahepatic activity. The Y90-RE dose needed to achieve at least 120 Gy or 150 Gy dose to the respective targeted lobe or segment was calculated using the MIRD model as recommended by the package insert of glass-based microspheres (Therasphere®, Boston Scientific, Marlborough, MA, USA) [25]. Using the calculated activity at the same catheter position as the technetium-99m macroaggregated albumin shunt study, Y90-RE was then performed approximately two weeks after the shunt study. Patients then underwent Y90 Bremsstrahlung single-photon emission CT immediately post-Y90-RE to confirm delivery of the prescribed activity to the targeted area and tumor.

### 2.3. Statistical Analysis

Primary outcome measures included overall survival (OS) and disease-free survival (DFS). OS was calculated using Kaplan–Meier estimation as months from the time of first Y90-RE until death or last known follow-up. Where applicable, OS curves for subcohorts were compared using the log-rank test. Median DFS in months was calculated using the Kaplan–Meier method and defined as the time in months from the date of the Y90-RE until the last imaging study showing no evidence of intrahepatic or extrahepatic disease.

To ensure at least two years of follow-up time after the Y90-RE procedure, records review was limited to 2018. Descriptive statistics for each demographic and clinical variable were reported. The univariate association of each variable was assessed for favorable response vs. unfavorable response and inside Milan criteria vs. outside Milan criteria, using the *χ*2 and Fisher's exact test for categorical covariates and Student's *t*-test for numerical covariates. Statistical significance was defined as *p* value < 0.05.

Because multicollinearity issues may cause a multivariate logistic regression model to yield unreliable parameter estimates [26], an elastic net regression model (penalized logistic regression model, equation (1)), fitted with a combination of the lasso and ridge penalty functions within a generalized logistic regression model [27, 28], was used to adjust for potential covariates and to ultimately assess which prognostic baseline characteristics are associated with a favorable response.

*y*_*i*_ ~ Bernoulli (*p*_*i*_) is as follows:

(1) $$\begin{array}{c} \underset{\beta_{0},\beta_{j}}{\min}\left[\frac{1}{2n}\overset{n}{\underset{i=1}{\sum }}{\left(\log\;it\left(p_{i}\right)-\beta_{0}-\overset{e}{\underset{j=1}{\sum }}\beta_{j}X_{ij}\right)}^{2}+\lambda \overset{e}{\underset{j=1}{\sum }}\left(\frac{1-\alpha }{2}\beta_{j}^{2}+\alpha \left|\beta_{j}\right|\right)\right], \end{array}$$

where 0 ≤ *α* ≤ 1, *λ* ≥ 0; *y*_*i*_ is the *n* × 1 vector of outcome values for *i* = 1 to *n* participants; *p*_*i*_ is the probability of outcome (i.e., favorable response); logit(*p*_*i*_)=*logp*_*i*_/1 − *p*_*i*_; *β*_0_ is the intercept; *β*_*j*_ are vectors of regression coefficients that correspond to **X**_*ij*_, *j* = 1 to *e* (the *n* × *e* matrix of standardized predictors). *α* controls the balance between the lasso (*α* = 1) and ridge (*α* = 0) penalties. *λ* represents the penalty parameter, with the degree of shrinkage increasing as *λ* increases for a given *α* value.

A cross-validation (CV) method was used to determine the optimal parameters *α* and *λ*. To attain a satisfactory true positive rate of selection [29], the models were tested over 100 times by repeating a 10-fold CV over a group of *α* and *λ* sequences. The combination of *α* and *λ* that yielded a minimum mean-squared error was then selected for further analysis. Any baseline characteristic variables with more than 40% missing values or a size less than 10% of the total cohort were excluded. Additionally, variables were scaled to have the same mean and standard deviation to achieve both similar importance in the penalized regressions modeling and to improve the comparability of coefficients for continuous and categorical predictors. The model was then executed for 50 continuous times with the optimal *α* and *λ*, resulting in the voting of variables. Baseline characteristic variables that were voted on at least 40 times were considered significant, and odds ratios were accordingly calculated from the reported mean of their coefficient estimations.

All statistical analyses were performed using JMP statistical software (JMP Pro, Version 13. SAS Institute Inc., Cary, North Carolina), *R* Statistical Software (version 4.0.3, *R* Foundation for Statistical Computing, Vienna, Austria) with the fit elastic net models found within the “glmnet” package [27], and Excel Office 365 (Microsoft Corporation, Redmond, Washington).

## 3. Results

### 3.1. Overall Study Cohort

From 2013–2018, 257 patients underwent 304 Y90-RE therapies for the treatment of HCC. A total of 135 patients met the inclusion criteria and underwent Y90-RE as bridging or downstaging therapy for their Y90-RE naïve HCC (Figure 1). The overall study cohort was predominantly composed of Caucasian males (57%) with a mean age of 65 ± 10 years of age and a liver disease etiology that was often associated with sequelae of hepatitis C virus (57%) and alcohol consumption (21%). Pre-Y90-RE liver/HCC directed therapies (e.g., TACE, surgical resection, and ablation) and systemic therapy (e.g., Sorafenib) were only present in 28% and 3% of the overall cohort, respectively. Y90-segmentectomy was performed in 70% of the overall cohort (Table 1).

The overall cohort's mean time from pre-Y90-RE cross-sectional imaging to Y90-RE was 2.3 ± 1.7 months. The cohort's HCC tumors were predominantly of the unilobar extent within a cirrhotic liver and were diagnosed by imaging using DCE CT/MRI (Table 2). The top three baseline symptoms at pretreatment evaluation were fatigue (21%), abdominal pain (18%), and ascites (10%) (Table 3). The mean baseline alpha-fetoprotein (AFP) tumor marker was 263 ± 526 ng/mL. Posttreatment analysis revealed an overall mean lung shunt fraction of 7 ± 4% with a mean calculated lung dose of 9 ± 6 Gy and a mean tumor dose of 651 ± 452 Gy and 188 ± 95 Gy during segmentectomy and lobar treatment cases, respectively.

### 3.2. Y90-RE Response and Liver Transplantation Outcomes

Out of the 135 patients in the eligible cohort, 42% were within MC and 56% were outside MC at baseline (Figure 1). Among the 59 patients who were within MC, eligibility was maintained in 83% and lost in 17% (significant baseline differences between these two subcohorts are highlighted in Table 4). Out of the 76 patients that were outside MC, eligibility was achieved in 42% and remained unchanged in 58%. Grouping the maintained and achieved MC groups, a cohort of 81 patients (60% of the total cohort) was deemed to have a favorable therapy response to Y90-RE. Grouping the unchanged and lost MC groups, a cohort of 54 patients (40% of the total cohort) was deemed to have an unfavorable therapy response to Y90-RE.

At the end of the follow-up period, 44% of the overall cohort was confirmed to be deceased, 23% was alive, either waiting for a LT or no longer LT eligible secondary to non-HCC-related reasons (e.g., new substance abuse, new personal wishes, and new medical comorbidities), and 18% was unaccounted for longer than >12 months. All 20 patients (15% of the overall cohort) who had received a LT were still alive, with the favorable response cohort demonstrating a higher incidence of LT than the unfavorable response cohort (16 (20%) vs. 4 (7%); *p* = 0.04). No difference in LT incidence rate was noted between the within and outside MC groups (11 (19%) vs. 9 (12%); *p* = 0.2). Including patients who received LT, the overall mean and median (with interquartile range (IQR)) overall survival were 24.0 ± 16.1 months and 22.1 months (25.4 months IRQ), respectively. Excluding those who received LT, the overall mean and median overall survival were, respectively, 21.7 ± 15.1 months and 19.8 months (24.3 months IQR). In the overall cohort, an mRECIST complete response immediately after Y90-RE was attained in 42% of patients, and the median disease-free survival of this cohort was 9.0 months (18.1 months IQR).

### 3.3. Within and Outside Milan Criteria: Baseline Demographics and Clinical Differences

No differences in terms of age, gender, ethnicity, and etiology of liver disease were found between the within and outside MC groups. The within MC group demonstrated a lesser percentage of liver directed procedures prior to Y90-RE (15% vs. 38%; *p* = 0.003) and a greater percentage of Y90-RE segmentectomy procedures (86% vs. 57%; *p* = 0.0001). Both groups were comprised of patients who were predominantly of ECOG score 0, ALBI grade 2, CP class A, and MELD 10. Of note, there were two patients in the within MC (and favorable response) group who were ECOG 3 secondary to a musculoskeletal injury resulting in limited self-care and confinement of >50% to a wheelchair. While the ECOG, MELD, and CP class were not significantly different between the two groups, the ALBI grade, AAPR, and BCLC stage were significantly different, with the within MC group exhibiting a more favorable ALBI grade, AAPR, and BCLC stage than the outside MC group (Table 1). For example, the differences in low vs. intermediate/high mortality ALBI grades were 22 (37%) and 37 (62%) in the within MC group and 8 (11%) and 68 (89%) in the outside MC group (*p* ≤ 0.0001). AAPR was lower in the outside MC group than in the inside MC group (0.33 ± 0.15 vs. 0.41 ± 0.15; *p* = 0.003). Differences between the very early/early vs. intermediate/advanced/terminal BCLC stages for the within and outside MC groups were, respectively, 41 (69%) and 18 (30%) vs. 15 (20%) and 61 (81%) (*p* ≤ 0.0001). The two BCLC *D* patients in the within MC (and favorable response) group were secondary to the aforementioned ECOG 3 status.

### 3.4. Within and Outside Milan Criteria: Baseline Imaging Characteristics

Patients in the within MC group had a higher incidence of baseline unilobar disease (95% vs. 57%; *p* ≤ 0.0001), smaller dominant tumor mean diameter (31 ± 12 mm vs. 44 ± 18 mm; *p* ≤ 0.0001), smaller total tumor cumulative mean diameter (32 ± 12 mm vs. 72 ± 27 mm; *p* ≤ 0.0001), and a lower incidence of ≥4 HCC tumors (0% vs. 37%; *p* ≤ 0.0001) (Table 2).

### 3.5. Within and Outside Milan Criteria: Baseline and Post-Y90-Re Laboratory and Symptom Adverse Events

The within MC group had higher levels of albumin (3.7 ± 0.5 g/dL vs. 3.5 ± 0.4 g/dL; *p* = 0.02) and lower levels of alkaline phosphatase (107 ± 53 U/L vs. 137 ± 91 U/L; *p* = 0.02) than the outside MC group. No significant differences in baseline symptoms were noted between the two groups. After Y90-RE therapy, the within MC group demonstrated a lower incidence of ascites (12% vs. 26%; *p* = 0.03), lower levels of alkaline phosphatase (124 ± 58 U/L vs. 161 ± 114; *p* = 0.02), and higher levels of albumin (3.6 ± 0.5 g/dL vs. 3.3 ± 0.6 g/dL; *p* = 0.004) than the outside MC group. There were no differences in the incidence of serious AE in symptoms or laboratory values between the two groups (Table 3 and Table 5).

### 3.6. Within and Outside Milan Criteria: Outcomes

The within MC group had a higher incidence of mRECIST complete/partial response (50 (85%) vs. 36 (47%); *p* ≤ 0.0001)), smaller dominant tumor mean diameter (27 ± 20 mm vs. 41 ± 26 mm; *p* = 0.02), smaller total tumor cumulative mean diameter (10 ± 22 mm vs. 53 ± 43 mm; *p* ≤ 0.0001), and a lower incidence of ≥4 HCC tumors (5% vs. 28%; *p* = 0.0004) than the outside MC group (Table 2). Between the within and outside MC groups, no difference in mean OS (25.7 ± 16.1 months vs. 22.7 ± 16.1 months) or median OS (25.4 months (28.1 months IQR) vs. 19.8 months (25.0 months IQR)) was observed (*p* = 0.3). When LT patients were excluded from analysis, this relationship persisted, with no difference in mean OS (25.7 ± 16.1 months vs. 20.5 ± 14.6 months) or median OS (25.4 months (28.0 months IQR) vs. 16.7 months (23.2 months IQR)) being observed between the within and outside MC groups (*p* = 0 .07). The overall median length of DFS after Y90-RE was 8.9 months (16.4 months IQR) for the within MC group and 9.4 months (23.4 months IQR) for the outside MC group.

### 3.7. Favorable and Unfavorable Response Cohorts: Baseline Demographics and Clinical Characteristics

The favorable response cohort demonstrated a greater amount of segmentectomy procedures (83% vs. 50%; *p* = 0.0001). Both the favorable and unfavorable response cohorts were comprised of patients who were predominantly of ECOG score 0, ALBI grade 2, CP class A, and MELD 10. While the ECOG status and MELD score were not different between the two cohorts, the favorable response cohort exhibited a more favorable ALBI grade, AAPR, CP class, and BCLC stage than the unfavorable response cohort (Table 1). For example, the differences in low vs. intermediate/high mortality ALBI grades were 25 (31%) and 56 (69%) in the favorable response cohort and 5 (9%) and 49 (91%) in the unfavorable response cohort (*p* = 0.002). AAPR was lower in the unfavorable response cohort than in the favorable response cohort (0.32 ± 0.15 vs. 0.39 ± 0.16; *p* = 0.007). Differences between good vs. moderate/advanced hepatic dysfunction CP classes were 75 (93%) and 6 (7%) in the favorable response cohort and 42 (78%) and 12 (22%) in the unfavorable response cohort (*p* = 0.01). Differences between the very early/early vs. intermediate/advanced/terminal BCLC stage for the favorable and unfavorable response cohorts were 48 (59%) and 31 (38%) vs. 8 (15%) and 46 (85%), respectively (*p* ≤ 0.0001). No difference in age, gender, ethnicity, etiology of liver disease, or incidence of pre-Y90-RE liver directed procedures was found between the unfavorable and favorable response cohorts.

### 3.8. Favorable and Unfavorable Response Cohorts: Baseline Imaging Characteristics

The favorable response cohort had a higher incidence of being diagnosed with DCE CT or MRI as opposed to requiring percutaneous tissue biopsy (94% vs. 74%; *p* = 0.003), smaller dominant tumor mean diameter (35 ± 15 mm vs. 44 ± 18 mm; *p* = 0.006), smaller total tumor cumulative mean diameter (43 ± 23 mm vs. 70 ± 30 mm; *p* ≤ 0.001), and a lower incidence of ≥4 HCC tumors (6% vs. 43%; *p* ≤ 0.001) (Table 2).

### 3.9. Favorable and Unfavorable Response Cohorts: Baseline and Post-Y90-Re Laboratory and Symptom Adverse Events

Patients in the favorable response cohort had a lower incidence of fatigue (16% vs. 30%; *p* = 0.04), lower incidence of abdominal pain (11% vs. 28%; *p* = 0.01), lower levels of alkaline phosphatase (111 ± 55 U/L vs. 143 ± 100 U/L; *p* = 0.04), and higher levels of albumin (3.7 ± 0.5 g/dL vs. 3.4 ± 0.4 g/dL; *p* = 0.002). After Y90-RE therapy, patients in the favorable response cohort demonstrated a lower incidence of ascites (11% vs. 33%; *p* = 0.001), lower levels of AFP tumor marker (77 ± 286 ng/mL vs. 452 ± 656 ng/mL; *p* = 0.002), lower levels of alkaline phosphatase (127 ± 55 U/L vs. 169 ± 130 U/L; *p* = 0.04), and higher levels of albumin (3.6 ± 0.5 g/dL vs. 3.1 ± 0.7; *p* ≤ 0.0001). There were no differences in the incidence of serious AE in symptoms or laboratory values between the two cohorts (Tables 3 and 5).

### 3.10. Favorable and Unfavorable Response Cohorts: Outcomes

**Figure 2:**
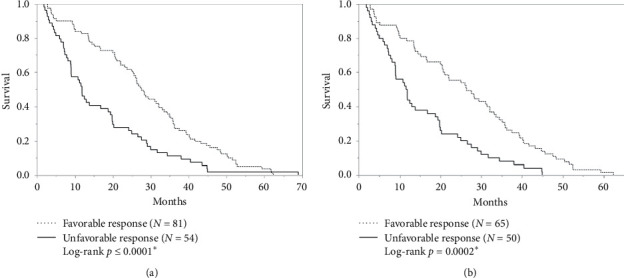
Overall survival for (a) complete cohort and (b) cohort censored to liver transplantation. In the complete cohort, the favorable response cohort had a longer median OS than the unfavorable response cohort (27.6 months vs. 11.8 months; *p* ≤ 0.0001). When liver transplantation was censored from the overall cohort, the favorable response cohort continued to have a longer median OS than the unfavorable response cohort (26.4 months vs. 11.8 months; *p* = 0.0002).

After Y90-RE therapy, patients in the favorable response cohort demonstrated a higher incidence of mRECIST complete/partial response (73 (90%) vs. 13 (25%); *p* ≤ 0.0001), smaller dominant tumor mean diameter (35 ± 15 mm vs. 44 ± 18 mm; *p* = 0.006), smaller total tumor cumulative mean diameter (44 ± 23 mm vs. 70 ± 30 mm; *p* ≤ 0.0001), and a lower incidence of ≥4 HCC tumors (6% vs. 43%; *p* ≤ 0.0001) - (Table 2). Compared to the unfavorable response cohort, the favorable response cohort exhibited a longer mean OS (28.7 ± 15.6 months vs. 16.9 ± 14.0 months; *p* ≤ 0.0001) and median OS (27.6 months (23.5 months IQR) vs. 11.8 months (18.5 months IQR)). Differences in mean and median OS remained after excluding patients with LT, with the favorable response cohort exhibiting a longer mean OS (26.7 ± 15.5 months vs. 16.9 ± 14.1 months; *p* = 0.0002) and median OS (26.4 months (24.9 months IQR) vs. 11.8 months (18.4 months IQR)) than the unfavorable response cohort (Figure 2). In patients who achieved an mRECIST complete response after Y90-RE, the overall median length of DFS after Y90-RE was 9.0 months (18.1 months IQR).

### 3.11. Favorable and Unfavorable Response Cohorts: Penalized Logistic Regression Analysis of the Baseline Characteristics

Eighteen baseline characteristics were associated with the successful bridging or downstaging to LT criteria in patients with HCC undergoing Y90-RE, as shown in Table 6. These prognostic factors were part of nine categories of clinical and imaging baseline characteristics, with some demonstrating positive while others negative prognostic value. Briefly, the significant categories of baseline characteristics were the method of HCC diagnosis, etiology of liver disease, lesion characteristics, affected hepatic lobe, ALBI grade, Child–Pugh Class, BCLC Stage, baseline symptoms, and baseline laboratory levels. The logistic regression coefficients and their corresponding odds ratio for each of the prognosticators are shown in Table 6.

## 4. Discussion

The use of bridging or downstaging therapies began as a result of the overwhelming disparity between the need for and supply of transplantable livers [30]. While TACE or radiofrequency ablation has historically been the most commonly used therapies for these endeavors [31], the role of Y90-RE in the management of patients with HCC has become further prominent in the past decade [32]. To date, conventional TACE remains the official first-line therapy for certain types of HCCs [33]. However, a growing body of literature has challenged this notion by suggesting that Y90-RE can provide similar or superior outcomes to TACE or radiofrequency ablation [10, 34–36]. Although Y90-RE has the potential to significantly impact waitlist times and posttransplant outcomes, the level of evidence in support of its use as a pre-LT therapy remains very low [37, 38]. Furthermore, variations in tumor response after Y90-RE remain poorly understood and can complicate a patient's treatment plan, especially for patients being evaluated for LT. To help clinicians have a better understanding of a patient's prognosis prior to Y90-RE therapy, this article explores the associations between clinical and imaging prognosticators and the successful bridging or downstaging to LT criteria in patients with HCC undergoing Y90-RE.

Using penalized logistic regression analysis, odds ratios were calculated for each of the eighteen clinical and imaging baseline prognosticators that were significant. From a practical perspective, these odds ratios can permit a clinician to calculate the odds of achieving a successful bridging or downstaging to LT criteria in patients undergoing Y90-RE therapy for HCC, by applying the odds ratio either in a single fashion whenever a covariable prognosticator is present (e.g., left lobe HCC disease) or in multiplicative fashion whenever a continuous numerical prognosticator is present (e.g., serum albumin level). Caution must be raised whenever the odds ratios of certain baseline characteristics (e.g., nausea and other liver disease etiologies) are being interpreted, for their calculation was based out of limited/small cohort sizes; thus, their prognostic importance was likely exaggerated by our logistic regression model.

While the challenges associated with the clinical management of patients with HCC have been extensively studied [39, 40], research evaluating the use of a patient's subjective clinical symptoms as a prognostic indicator for outcomes remains limited. In general, because most patients with HCC are diagnosed at an advanced stage [41], a triad of right upper quadrant abdominal pain, palpable mass, and weight loss is often present [42]. These symptoms are often associated with the severity of tumor burden, especially abdominal pain, whose presence has been associated with tumor visceral involvement [43]. In this study, however, none of the patients had baseline tumoral involvement of the macrovascular, extrahepatic, or visceral structures. Nevertheless, the absence of certain baseline symptoms such as fatigue and abdominal pain was associated with the successful bridging or downstaging to LT eligibility. An explanation for this may be the unfavorable response cohort's worse hepatic function and tumor burden, which in addition to debilitating the liver's function, it may have also indirectly aggravated the liver capsule and abdominal viscera without invading it. Interestingly, the unfavorable response cohort had a higher incidence of HCC diagnosis driven by tissue sampling (instead of by DCE CT/MRI), which was too found to be a negative prognosticator. Since tumor seeding was not observed in post-Y90-RE imaging, this finding may be only indicative of a more biologically complex HCC that was unable to be conclusively diagnosed via DCE CT/MRI. An additional nonclinical baseline characteristic that had a negative prognostic value was HBV-associated cirrhosis. This was surprising to the authors, for patients at the study's institution with known chronic HBV are placed on antiviral treatment and those with HBV-associated cirrhosis are placed in an imaging surveillance program that strives to improve morbidity and mortality by conducting screening for HCC [44].

Lesion-wise, a greater degree of tumor burden was found to be negatively associated with a favorable response. While some of these prognosticators were expected (e.g., largest lesion diameter, total cumulative tumor diameter, presence of ≥4 viable HCC masses, multilobar disease), others, such as the presence of left lobe HCC disease, were not. The ability of left lobe HCC disease to act as a positive prognosticator for attaining a favorable response may be driven in part by the relatively smaller volume and decreased incidence of vascular variations [45] within a left hepatic lobe, for this combination of a smaller and easier to reach targeted volume can permit a technically easier delivery Y90 particles at high radiation doses. Nevertheless, no study to date has evaluated the clinical outcomes of HCC patients in relation to the disease site to be treated with Y90-RE. In the surgical literature, however, left hepatic lobe HCC disease has been associated with worse clinical outcomes after surgical intervention [46], suspected to be driven in part by the greater difficulty in attaining negative surgical margins.

As a result of the greater tumor burden, the unfavorable response cohort exhibited worse liver function markers (e.g., ALBI grade, AAPR, CP, albumin, and alkaline phosphatase) and BCLC stage at baseline than the favorable response group. Nevertheless, the penalized logistic regression analysis revealed that only ALBI grade of 2, CP classes A and B, BCLC stage A and B, and serum albumin levels were significant in terms of prognostic value. Close inspection of the magnitude of the odds ratio for each of these prognostic variables suggests that lower ALBI grade, lower CP class, lower BCLC stage, and normal/higher albumin serum levels are all associated with the successful bridging or downstaging to LT eligibility with Y90-RE therapy. As predicted by the more favorable AAPR, ALBI grade, CP Class, and BCLC stage at baseline, the favorable response cohort exhibited a longer OS than the unfavorable response cohort. By extension, these results highlight the association between successfully downstaging or bringing to LT eligibility and achieving a more favorable OS—an association previously described [47] but only recently suggested to be more important than freedom from tumor progression in patients undergoing Y90-RE [48].

In concordance with the posttherapy outcomes described in the literature [37, 38], nearly half (42%) of the overall cohort observed an mRECIST complete response after Y90-RE. While over 60% of the overall cohort was either able to maintain or achieve MC eligibility, a small number of patients (17% of the within MC at baseline cohort) lost their MC eligibility after Y90-RE. This finding highlights the risk and importance associated with carefully selecting HCC patients for Y90-RE bringing/downstaging, for the improper patient and/or treatment selection can result in loss of opportunity to receive curative treatment. In this study, only 15% of the overall cohort was able to attain LT by the time of the study's data tabulation. This quantity of patients receiving LT was relatively low, even when compared to published conservative incidence rates of LT [49]. An explanation for this is that HCC-related disease was the main driver in the decision to classify the included patients as potential LT candidates. In other words, non-HCC-related issues (e.g., substance abuse status and personal wishes) that either were developed or were not appropriately addressed at the time of or after MTB evaluation limited the ability of this study's patients to truly achieve their LT potential. This limitation, in addition to the observed incidence of LT among patients in the unfavorable response cohort, suggested a degree of clinical practice and cohort heterogeneity that was difficult to capture within the data of this study. Nevertheless, the favorable response cohort exhibited a significantly higher incidence of LT attainment than the unfavorable response cohort—an observation concordant with published literature suggesting that imaging response after locoregional therapy (i.e., downstaging and/or bridging achievability) is a surrogate for tumor biology and prognosticator for LT attainment and outcomes [50, 51].

Limitations of this study include its single-center retrospective nature and the relatively small sample size for some of the baseline characteristics. The tumor board's selection criteria for the cohort as a potential LT candidate were heavily based on HCC-related characteristics, which at times resulted in non-HCC-related characteristics limiting the patient's ability to fully achieve their LT potential. At the study's institution, Y90-RE has gained significant traction as one of the early HCC therapies to be considered, with TACE and radiofrequency ablation having a decreasing but still present role. While the Y90-RE techniques were not standardized and often varied to fit the patient's tumor(s) size and distribution, all patients underwent Y90-RE with curative intent, with post-Y90-RE data analysis demonstrating that all tumors received ablative doses with minimal incidence of adverse events (none of which were severe/life-threatening). Lastly, differences in patient selection criteria, particularly regarding hepatic function, exist between institutions and should be recognized prior to the application of these prognosticators on patient populations.

## 5. Conclusion

The successful bridging or downstaging to LT criteria in patients undergoing Y90-RE therapy for HCC is associated with a lower ALBI grade, lower CP class, lower BCLC stage, HCC diagnosis with DCE CT or MRI, normal/higher albumin levels, a more limited tumor burden, left lobar disease, and the absence of HBV-associated cirrhosis, baseline abdominal pain, or fatigue.

## Figures and Tables

**Table 1:** Baseline demographics and clinical characteristics.

Characteristic	All cohort	Outside Milan criteria	Inside Milan criteria	*p* value	Unfavorable response	Favorable response	*p* value
Total number of patients	*n* = 135	*n* = 76	*n* = 59		*n* = 54	*n* = 81	
Age, mean	65 ± 10	66 ± 8	64 ± 12	0.1	66 ± 8	65 ± 11	0.5
Gender, female	29 (22%)	15 (20%)	14 (24%)	0.5	11 (20%)	18 (22%)	0.5

*Ethnicity*							
Caucasian	77 (57%)	44 (58%)	33 (56%)	0.3	36 (67%)	41 (51%)	0.1
African American	39 (29%)	20 (26%)	19 (32%)		13 (24%)	26 (32%)	
Asian	10 (7%)	6 (8%)	4 (7%)		3 (6%)	7 (8%)	
Hispanic	2 (2%)	0 (0%)	2 (3%)		1 (2%)	1 (1%)	
Other	7 (5%)	6 (8%)	1 (2%)		1 (2%)	6 (7%)	

*Pre-Y90-RE HCC therapies*							
Liver directed procedures	38 (28%)	29 (38%)	9 (15%)	0.003^*∗*^	19 (35%)	19 (23%)	0.09
Systemic therapies	4 (3%)	7 (11%)	0 (0%)	1	5 (9%)	2 (2%)	0.09
*Y90-RE segmentectomy*	94 (70%)	43 (57%)	51 (86%)	0.0001^*∗*^	27 (50%)	67 (83%)	0.0001^*∗*^

*Etiology of liver disease*							
Hepatitis B virus	16 (12%)	11 (14%)	5 (8%)	0.2	8 (15%)	8 (10%)	0.3
Hepatitis C virus	77 (57%)	43 (57%)	34 (58%)	0.5	32 (59%)	45 (56%)	0.4
EtOH abuse	29 (21%)	15 (20%)	14 (24%)	0.5	12 (22%)	17 (21%)	0.7
Nonalcoholic steatohepatitis	16 (12%)	12 (16%)	4 (7%)	0.09	7 (13%)	9 (11%)	0.5
Hemochromatosis	1 (1%)	1 (1%)	0 (0%)	0.6	1 (2%)	0 (0%)	0.4
Other	22 (16%)	11 (14%)	11 (%)	0.3	10 (18%)	12 (14%)	0.4

*ECOG performance status*							
0	96 (71%)	55 (72%)	41 (69%)	0.6	36 (67%)	60 (74%)	0.3
1	34 (25%)	20 (26%)	14 (24%)		16 (30%)	18 (22%)	
2	3 (2%)	1 (1%)	2 (3%)		2 (4%)	1 (1%)	
3	2 (1%)	0 (0%)	2 (3%)		0 (0%)	2 (2%)	

*ALBI grade*							
1	30 (22%)	8 (11%)	22 (37%)	0.0002^*∗*^	5 (9%)	25 (31%)	0.002^*∗*^
2	98 (73%)	64 (84%)	34 (58%)		46 (85%)	52 (64%)	
3	7 (5%)	4 (5%)	3 (5%)		3 (6%)	4 (5%)	
*MELD, mean*	10 ± 3	10 ± 3	10 ± 3	1	10 ± 3	10 ± 3	0.7
*AAPR, mean*	0.36 ± 0.16	0.33 ± 0.15	0.41 ± 0.15	0.003^*∗*^	0.32 ± 0.15	0.39 ± 0.16	0.007^*∗*^

Child–Pugh class							
A	117 (87%)	64 (84%)	53 (90%)	0.2	42 (78%)	75 (93%)	0.01^*∗*^
B	18 (13%)	12 (16%)	6 (10%)		12 (22%)	6 (7%)	
C	0 (0%)	0 (0%)	0 (0%)		0 (0%)	0 (0%)	

*BCLC stage grade*							
0	5 (4%)	0 (0%)	5 (8%)	0.0001^*∗*^	1 (2%)	4 (5%)	0.0001^*∗*^
A	51 (38%)	15 (20%)	36 (61%)		7 (13%)	44 (54%)	
B	40 (30%)	40 (53%)	0 (0%)		28 (52%)	12 (15%)	
C	37 (27%)	21 (28%)	16 (27%)		18 (33%)	19 (23%)	
D	2 (1%)	0 (0%)	2 (3%)		0 (0%)	2 (2%)	

The symbol ^*∗*^indicates a significant *p* value. *Y90‐RE* = yttrium‐90 radioembolization; *HCC* = hepatocellular carcinoma; *ECOG* = Eastern Cooperative Oncology Group; *ALBI* = albumin‐bilirubin; *MELD* = model for end‐stage liver disease; *AAPR* = Albuminato-alkaline phosphatase ratio; *BCLC* = Barcelona Clinic Liver Cancer.

**Table 2:** Baseline tumor characteristics.

Characteristic	All cohort	Outside Milan criteria	Inside Milan criteria	*p* value	Unfavorable response	Favorable response	*p* value
Time from Pre-Y90-RE imaging to Y90-RE, mean months	2.3 ± 1.7	2.3 ± 1.1	69 ± 67	0.9	2.5 ± 1.3	2.1 ± 1.9	0.2
Method of diagnosis, imaging	116 (86%)	63 (83%)	53 (91%)	0.1	40 (74%)	76 (94%)	0.003^*∗*^
Hepatic cirrhosis	119 (88%)	66 (87%)	53 (90%)	0.4	46 (85%)	73 (90%)	0.3

*Affected hepatic lobe*							
Right lobe	75 (55%)	6 (8%)	18 (31%)	0.03^*∗*^	3 (6%)	21 (26%)	0.06
Left lobe	24 (18%)	37 (49%)	38 (64%)		23 (43%)	52 (64%)	
Both lobes (multilobar)	36 (27%)	33 (43%)	3 (5%)		28 (52%)	8 (10%)	

*Tumor characteristics*							
Largest tumor diameter, mean (mm)	39 ± 17	44 ± 18	31 ± 12	≤0.0001^*∗*^	44 ± 18	35 ± 15	0.006^*∗*^
2nd largest tumor diameter, mean (mm)	21 ± 10	22 ± 10	11 ± 6	0.1	22 ± 10	20 ± 10	0.3
3rd largest tumor diameter, mean (mm)	15 ± 6	15 ± 6	8 ± 3	0.06	15 ± 6	13 ± 5	0.2
Total tumor cumulative diameter, mean (mm)	54 ± 29	72 ± 27	32 ± 12	≤0.0001^*∗*^	70 ± 30	44 ± 23	0.0001^*∗*^
Patients with ≥4 HCC masses	28 (21%)	28 (37%)	0 (0%)	≤0.0001^*∗*^	23 (43%)	5 (6%)	0.0001^*∗*^

*Post Y90-RE tumor characteristics*							
mRECIST response							
Complete response	56 (42%)	14 (18%)	42 (71%)	≤0.0001^*∗*^	3 (6%)	53 (65%)	0.0001^*∗*^
Partial response	30 (22%)	22 (29%)	8 (14%)		10 (19%)	20 (25%)	
Stable disease	34 (25%)	30 (39%)	4 (7%)		26 (48%)	8 (10%)	
Progression of disease	15 (11%)	10 (13%)	5 (8%)		15 (28%)	0 (0%)	

*Tumor characteristics*							
Largest tumor diameter, mean (mm)	38 ± 26	41 ± 26	27 ± 20	0.02^*∗*^	44 ± 18	35 ± 15	0.006^*∗*^
2nd largest tumor diameter, mean (mm)	22 ± 11	23 ± 11	15 ± 5	0.01^*∗*^	22 ± 10	20 ± 10	0.4
3rd largest tumor diameter, mean (mm)	15 ± 8	15 ± 7	12 ± 8	0.6	15 ± 6	13 ± 5	0.2
Total tumor cumulative diameter, mean (mm)	34 ± 41	53 ± 43	10 ± 22	≤0.0001^*∗*^	70 ± 30	44 ± 23	≤0.0001^*∗*^
Patients with ≥4 HCC masses	24 (18%)	21 (28%)	3 (5%)	0.0004^*∗*^	23 (43%)	5 (6%)	≤0.0001^*∗*^

The symbol ^*∗*^ indicates a significant value. MC = Milan criteria; ECOG = Eastern Cooperative Oncology Group; ALBI = albumin-bilirubin; BCLC = Barcelona Clinic Liver Cancer.

**Table 3:** Clinical and laboratory characteristics.

Baseline symptoms	Outside Milan criteria	Inside Milan criteria	*p* value	Unfavorable response	Favorable response	*p* value
Encephalopathy	3 (4%)	0 (0%)	0.2	3 (6%)	0 (0%)	0.6
Ascites	10 (13%)	3 (5%)	0.09	9 (17%)	4 (5%)	0.02^*∗*^
Fatigue	20 (26%)	9 (15%)	0.09	16 (30%)	13 (16%)	0.048^*∗*^
Abdominal pain	15 (20%)	9 (15%)	0.3	15 (28%)	9 (11%)	0.01^*∗*^
Nausea	3 (4%)	1 (1%)	0.4	1 (2%)	3 (4%)	0.5
Vomiting	1 (1%)	1 (2%)	0.7	1 (2%)	1 (1%)	0.6
Anorexia	3 (4%)	1 (2%)	0.4	2 (4%)	2 (2%)	0.5
Constipation	4 (5%)	1 (2%)	0.3	3 (6%)	2 (2%)	0.3
Fever	0 (0%)	0 (0%)	1	0 (0%)	0 (0%)	1

*Baseline laboratory levels*						
INR	1.1 ± 0.1	1.1 ± 0.1	0.9	1.1 ± 0.1	1.1 ± 0.1	1
AFP (ng/mL)	227 ± 453	314 ± 614	0.4	308 ± 513	233 ± 532	0.5
Aspartate transaminase (U/L)	62 ± 45	52 ± 38	0.2	60 ± 37	56 ± 46	0.6
Alkaline phosphatase (U/L)	137 ± 91	107 ± 53	0.02^*∗*^	143 ± 100	111 ± 55	0.04∗
Alanine transaminase (U/L)	51 ± 46	45 ± 37	0.4	50 ± 40	48 ± 44	0.7
Total bilirubin (mg/dL)	0.9 ± 0.4	1.0 ± 0.6	0.2	1.0 ± 0.5	0.9 ± 0.5	0.9
Creatinine (mg/dL)	1.1 ± 0.9	1.1 ± 0.9	0.9	1.0 ± 0.5	1.1 ± 1.1	0.5
Albumin (g/dL)	3.5 ± 0.4	3.7 ± 0.5	0.02^*∗*^	3.4 ± 0.4	3.7 ± 0.5	0.002^*∗*^
Sodium (mEq/L)	138 ± 2.9	138 ± 2.8	0.2	138 ± 3.0	137 ± 2.8	0.4

*Post Y90-RE symptoms*						
Encephalopathy	0 (0%)	1 (2%)	0.4	0 (0%)	1 (1%)	0.6
Ascites	20 (26%)	7 (12%)	0.03^*∗*^	18 (33%)	9 (11%)	0.001^*∗*^
Fatigue	29 (38%)	15 (25%)	0.08	20 (37%)	24 (30%)	0.2
Abdominal pain	25 (33%)	16 (27%)	0.3	18 (33%)	23 (28%)	0.3
Nausea	11 (14%)	10 (17%)	0.5	11 (20%)	10 (12%)	0.1
Vomiting	2 (3%)	4 (7%)	0.2	2 (4%)	4 (5%)	0.6
Anorexia	9 (12%)	6 (10%)	0.5	6 (11%)	9 (11%)	0.6
Constipation	4 (5%)	0 (0%)	0.1	2 (4%)	2 (2%)	0.5
Fever	1 (1%)	0 (0%)	0.6	0 (0%)	1 (1%)	0.6

*Post Y90-RE laboratory levels*						
INR	1.2 ± 0.4	1.2 ± 0.2	0.4	1.3 ± 0.4	1.2 ± 0.5	0.5
AFP (ng/mL)	311 ± 576	134 ± 392	0.9	452 ± 656	77 ± 286	0.002^*∗*^
Aspartate transaminase (U/L)	65 ± 45	51 ± 37	0.7	65 ± 47	54 ± 38	0.2
Alkaline phosphatase (U/L)	161 ± 114	124 ± 58	0.02^*∗*^	169 ± 130	127 ± 55	0.04^*∗*^
Alanine transaminase (U/L)	47 ± 43	39 ± 28	0.2	43 ± 32	44 ± 40	0.8
Total bilirubin (mg/dL)	1.1 ± 0.7	1.1 ± 0.7	0.8	1.2 ± 0.7	1.1 ± 0.7	0.7
Creatinine (mg/dL)	1.1 ± 1.1	1.1 ± 0.9	0.9	1.0 ± 0.7	1.1 ± 1.2	0.7
Albumin (g/dL)	3.3 ± 0.6	3.6 ± 0.5	0.004^*∗*^	3.1 ± 0.7	3.6 ± 0.5	≤0.0001^*∗*^
Sodium (mEq/L)	136 ± 2.7	137 ± 3.3	0.2	136 ± 2.8	137 ± 3.1	0.06

The symbol ^*∗*^ indicates a significant value. INR = international normalized ratio; AFP = alpha-fetoprotein. AFP = alpha-fetoprotein; Y90‐RE = yttrium‐90 radioembolization.

**Table 4:** Baseline clinical laboratory tumor characteristics of the inside Milan criteria patients: significant differences only.

Characteristic	All inside MC eligibility	Lost MC eligibility	Maintained MC eligibility	*p* value
Total number of patients	*n* = 59	*n* = 10	*n* = 49	
*ECOG performance status*				
0	41 (69%)	4 (40%)	37 (76%)	0.04^*∗*^
1	14 (24%)	5 (50%)	9 (18%)	
2	2 (3%)	1 (10%)	1 (2%)	
3	2 (3%)	0 (0%)	2 (4%)	

*ALBI grade*				
1	22 (37%)	1 (10%)	21 (43%)	0.049^*∗*^
2	34 (58%)	9 (90%)	25 (51%)	
3	3 (5%)	0 (0%)	3 (6%)	

*BCLC stage grade*				
0	5 (8%)	1 (10%)	4 (8%)	0.04^*∗*^
A	36 (61%)	3 (30%)	33 (67%)	
B	0 (0%)	0 (0%)	0 (0%)	
C	16 (27%)	6 (60%)	10 (21%)	
D	2 (3%)	0 (0%)	2 (4%)	

*Baseline symptoms*				
Fatigue	9 (15%)	4 (40%)	5 (10%)	0.03^*∗*^
Abdominal pain	9 (15%)	5 (50%)	4 (8%)	0.005^*∗*^

*Baseline laboratory levels*				
Albumin (g/dL)	3.7 ± 0.5	3.3 ± 0.4	3.8 ± 0.5	0.003^*∗*^

The symbol ^*∗*^ indicates a significant value. MC = Milan criteria; ECOG = Eastern Cooperative Oncology Group; ALBI = albumin-bilirubin; BCLC = Barcelona Clinic Liver Cancer.

**Table 5:** Adverse events status after Y90-RE, per Common Terminology Criteria for Adverse Events version 5.0.

Characteristic	Outside Milan criteria		Inside Milan criteria		*p* value	Unfavorable response		Favorable response		*p* value
Clinical symptoms present	Grade 1/2	Grade 3/4	Grade 1/2	Grade 3/4		Grade 1/2	Grade 3/4	Grade 1/2	Grade 3/4	
Encephalopathy	0 (0%)	0 (0%)	1 (2%)	0 (0%)	1	0 (0%)	0 (0%)	1 (1%)	0 (0%)	1
Ascites	20 (26%)	0 (0%)	5 (8%)	2 (3%)	0.06	18 (33%)	0 (0%)	7 (9%)	2 (2%)	0.1
Fatigue	28 (36%)	1 (1%)	15 (25%)	0 (0%)	0.7	19 (25%)	1 (2%)	24 (9%)	0 (0%)	0.5
Abdominal pain	24 (22%)	1 (1%)	15 (25%)	1 (2%)	0.6	16 (29%)	2 (4%)	23 (28%)	0 (0%)	0.2
Nausea	9 (12%)	2 (3%)	10 (17%)	0 (0%)	0.3	9 (17%)	2 (4%)	10 (12%)	0 (0%)	0.3
Vomiting	2 (3%)	0 (0%)	4 (6%)	0 (0%)	1	2 (4%)	0 (0%)	4 (5%)	0 (0%)	1
Anorexia	8 (10%)	1 (1%)	6 (10%)	0 (0%)	0.6	5 (9%)	1 (2%)	9 (11%)	0 (0%)	0.4
Constipation	4 (5%)	0 (0%)	0 (0%)	0 (0%)	1	2 (4%)	0 (0%)	2 (2%)	0 (0%)	1
Fever	1 (1%)	0 (0%)	0 (0%0	0 (0%)	1	0 (0%)	0 (0%)	1 (1%)	0 (0%)	1

	Inside Milan criteria		Outside Milan criteria		*p* value	Favorable response		Unfavorable response		*p* value
Laboratory levels	Grade 1/2	Grade 3/4	Grade 1/2	Grade 3/4		Grade 1/2	Grade 3/4	Grade 1/2	Grade 3/4	

INR	21 (28%)	1 (1%)	13 (22%)	1 (2%)	0.6	13 (24%)	1 (2%)	21 (26%)	1 (1%)	0.6
Aspartate transaminase	46 (61%)	3 (4%)	29 (49%)	1 (2%)	0.5	33 (61%)	2 (4%)	42 (52%)	2 (2%)	0.6
Alkaline phosphatase	49 (64%)	1 (1%)	28 (47%)	0 (0%)	0.6	35 (65%)	1 (%)	42 (52%)	0 (0%)	0.5
Alanine transaminase	15 (20%)	1 (1%)	13 (22%)	0 (0%)	0.6	9 (16%)	0 (0%)	19 (23%)	1 (1%)	0.7
Total bilirubin	24 (21%)	1 (1%)	23 (38%)	1 (2%)	0.7	21 (39%)	0 (0%)	28 (32%)	2 (2%)	0.3
Creatinine	10 (13%)	1 (1%)	7 (12%)	1 (2%)	0.7	8 (15%)	0 (0%)	9 (11%)	2 (2%)	0.3
Albumin	41 (54%)	3 (4%)	24 (41%)	0 (0%)	0.3	23 (61%)	3 (5%)	32 (39%)	0 (0%)	0.1
Sodium	31 (41%)	0 (0%)	21 (36%)	0 (0%)	1	24 (44%)	0 (0%)	28 (35%)	0 (0%)	1

INR = international normalized ratio.

**Table 6:** Penalized logistic regression analysis of the significant baseline characteristics.

Significant predictors	Favorable response	
	*β*	Exp (*β*)
HCC diagnosis by biopsy	−0.402	0.669
*Etiology of liver disease*		
Hepatitis B virus	−0.244	0.784
Other	−0.052	0.949

*Lesion characteristics*		
Largest lesion diameter, in mm	−0.003	0.997
Total tumor cumulative diameter, in mm	−0.005	0.995
Presence of ≥4 viable HCC masses	−0.314	0.730

*Affected hepatic lobe*		
Right lobe disease	−0.434	0.648
Left lobe disease	0.311	1.365
Multilobar disease	−0.398	0.671

*ALBI grade*		
Grade 2	−0.094	0.911

*Child–Pugh class*		
Class A	0.171	1.186
Class B	−0.171	0.843

*BCLC stage*		
Stage A	0.320	1.377
Stage B	−0.382	0.682

*Baseline symptoms*		
Fatigue	−0.032	0.969
Abdominal pain	−0.336	0.715
Nausea	0.215	1.240

*Baseline laboratory levels*		
Albumin (g/dL)	0.293	1.340

*β* = regression coefficient; exp (*β*) = odds ratio. *HCC* = hepatocellular carcinoma; *ALBI* = albumin-bilirubin; *BCLC* = Barcelona Clinic Liver Cancer.

## Data Availability

Anonymized clinical data can be made available upon request by the corresponding author.

## Abbreviations

AE: Adverse events
AAPR: Albumin-to-alkaline phosphatase ratio
ALBI: Albumin-bilirubin grade
AASLD: American Association for the Study of Liver Diseases
BCLC: Barcelona Clinic Liver Cancer Stage
CP: Child–Pugh class
CV: Cross-validation
DCE: Dynamic contrast-enhanced
DFS: Disease-free survival
ECOG: Eastern Cooperative Oncology Group score
HCC: Hepatocellular carcinoma
IQR: Interquartile range
LT: Liver transplantation
MELD: Model for end-stage liver disease
MC: Milan criteria
MTB: Multidisciplinary tumor board
mRECIST: Modified Response Evaluation Criteria in Solid Tumors
OS: Overall survival
TACE: Transarterial chemoembolization
Y90-RE: Yttrium-90 radioembolization.
